#  Small Duplication of HPRT 1 Gene May Be Causative For Lesh-Nyhan Disease in Iranian Patients 

**Published:** 2015

**Authors:** Razieh BOROUJERDI, Mohsen SHARIATI, Hosein NADDAFNIA, Hojatolah REZAEI

**Affiliations:** 1 Counselor in Welfare Organization of Qom, Iran; 2 Technical Corresponding in Pouya, Genetic Counseling Clinic, Qom, Iran; 3 Islamic Azad University Science and Research Branch, Tehran, Iran; 4 Counselor in Arman Genetic Counseling Clinic, Babol, Iran

**Keywords:** Lesch-Nyhan syndrome, HPRT gene, Purine metabolism, Prenatal diagnosis.

## Abstract

Deficiency of hypoxanthine-guanine phosphoribosyltransferase (HGPRT) is a rare inborn error of purine metabolism and is characterized by uric acid overproduction along with a variety of neurological manifestations that depend on a degree of the enzymatic deficiency. Inheritance of HPRT deficiency is X-linked recessive; thus, males are generally more affected and heterozygous females are carriers (usually asymptomatic). Human HPRT is encoded by a single structural gene on the long arm of the X chromosome at Xq26. More than 300 mutations in the HPRT1 gene have been detected. Diagnosis can be based on clinical and biochemical findings as well as enzymatic and molecular testing. Molecular diagnosis is the best way as it allows for faster and more accurate carrier and prenatal diagnosis. In this report, a new small duplication in the HPRT1 gene was found by sequencing, which has yet to be reported.

## Introduction

Hypoxanthine-guanine phosphoribosyltransferase (HGPRT) is a transferase enzyme that catalyzes conversion of hypoxanthine into inosine monophosphate (IMP); and guanine to guanosine monophosphate (GMP) (1). This enzyme provide cells with alternatives to the energy-expensive de novo synthesis of nucleotides and plays a critical role in the maintenance of intracellular purine nucleotide pools in cells that have decreased ability to synthesize new nucleotides (e.g. erythrocytes) (2). A deficiency of HGPRT in males results in a spectrum of disease, the severity of which is dependent upon the extent of the deficiency. A complete deficiency of the enzyme is associated with the Lesch-Nyhan syndrome (LNS) (incidences of 1 in 100,000), while partial deficiency is associated with gout (incidences 1 in 200 among males with gout) (3). LNS was first described by M. Lesch and W. Nyhan in 1964 as a complete deficiency of activity reported in two brothers (4) and clinically associated with neurologic dysfunction, cognitive and behavioral disturbances including self-mutilation, and uric acid overproduction (hyperuricemia). Most patients with LNS usually expire in childhood from renal failure and infection; and survival into second decade of life is known but rare (5). Sudden and unexpected deaths have respiratory rather than cardiogenic origins (6). Neurological symptoms affect the motor sphere and the cognitive and behavioral aspects (7). HGPRT is encoded in humans via the HPRT1 gene (size 42.5 kbp) (8) on the X chromosome in the region q26-q27 with only one transcript (9) and consists of 9 exons with a coding sequence of 654 bp (10). There are high degrees of heterogeneity in type and location within the gene (7). At least 400 different mutations in the HPRT coding parts of exons 1 to 9 are specified and mutations of substitution, frame shift, micro deletion, macro deletion, insertion, and splicing errors have been described as the cause of HGPRT deficiency (11, 12). However, about 30% of patients’ mothers are not somatic carriers; these patients probably carry de novo mutations due to a germinal cell mutation that requires genomic DNA sequencing (7). Here, we report a new small duplication that was detected by sequencing the entire HPRT1 gene in an Iranian family. 

## Case report

A woman (Consultand) had a mentally retarded brother who expired 15 years ago, was referred to the Medical Genetics Center. The patient’s parents were unrelated and there was a similar disease in her uncle’s son. The patient also had three uncles who expired under one year of age. 

**Fig 1:**
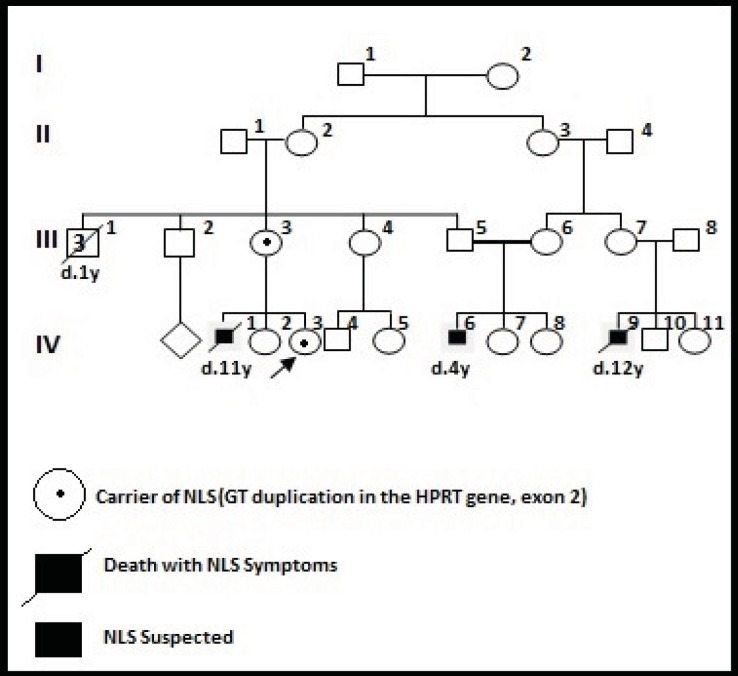
Consultand family pedigree

The patient’s uncle was married to a cousin and her uncle’s wife has a nephew with a similar disease who expired at 12 years of age (Figure 1).

According to the observations of the parents, the proband was quiet, hypotonic with neuro-developmental delays from birth. Metabolic and ultrasound screening was normal. The clinical findings were not shown until three years of age. From this time, the child started to say words and exhibited self-mutilation behavior that started with biting his clothes and chewing his fingers. He (brother of Consultand) has also had other clinical aspects of gastrointestinal defects such as constipation, kidney stones (staghorn calculus), hematuria, and finally expired due to a heart problem. 

Based on these findings LNS was considered. Identification of mutations has been performed at the RNA and DNA levels. Sequencing genomic DNA of the HPRT gene offers the possibility of direct diagnostic analysis independent on the expression of the mature HPRT mRNA (13). Accordingly, DNA was extracted and direct DNA sequencing was performed with dye terminators in her mother (molecular genetic analysis was performed by the Center for Nephrology and Metabolic Disorder Deutschland/Germany). The fragments were separated by capillary electrophoresis (ABI Prism 3130). All coding regions (exons) and the bordering invariant splice junction were sequenced for a total of 7,931 bp. The interpretation of the sequence data follows GenBank entry 00194. The c.105_106 duplication (GT) in exon 2 (codon 36) was found. The mutation is not yet in the database version available to us [especially PubMed indexed for MEDLINE (PMID), Single Nucleotide Polymorphism indexed for MEDLINE (SNPID)], but its pathogenic relevance is obvious as it causes a protein truncation in exon 3 (codon 16, ATC). 

Replication slippage is a commonly observed DNA replication error that is often facilitated by a repetitive sequence (14) and leads to short sequence duplications or deletions and if the mutation occurs in a coding region, it could produce abnormal proteins and leads to diseases (15). In fact, the mother (III-3) was heterozygous for this mutation. This mutation was studied in the consultand (IV-3) and she was like her mother. Therefore, the risk of the recurrence of LNS for her sons was raised to 50%. If the mutation is identified in NLS suspected individuals (IV-6), it may be concluded that the origin of the mutation in I-1 or I-2. Therefore, clinical examinations and further tests are required to prove NLS in this family. 

## Discussion

Since the inheritance of HPRT deficiency is X-linked recessive, males are generally. 

More affected and heterozygous female are the carriers. For families with an affected person, carrier diagnosis is important issue. Female carriers cannot be detected without the help of a laboratory since they are usually asymptomatic (16). However, their mosaicism in terms of HPRT activity, such diagnosis is not infallible (17) and molecular methods allow for faster and more accurate carrier diagnosis (18). Therefore, with the approval of the carriers, prenatal diagnosis can be performed by amniocentesis at about 15–18 weeks of gestation, or chorionic villus sampling (CVS) at about 10–12 weeks gestation for the known disease-causing mutation (19, 20). 

Since more than 300 mutations in this gene have been identified, there is also the possibility of identifying a new mutation. The direct DNA sequencing is one of the best ways to identify new mutations as many new mutations have been previously identified in this way as exampled in Gibbs et al (21) and Mak et al (22). 

In this family, detection of duplication mutations in exon-2 HPRT1 gene in the patient’s sister and this type of mutation is a frame-shift mutation, it is thought that the patient had a severe loss of enzyme activity. This loss could be the reason for the severity of the illness and the inability to survive into the early second decade of life. Thus, prenatal diagnosis must be performed to identify the affected or carriers fetuses in males and females, respectively. 

Despite the lack of complete and reliable treatment due to a lack of knowledge for all aspects of clinical and metabolic effects, accurate and timely diagnosis of deadly diseases such as LNS due to clinical follow-up can be effective in reducing the severity of diseases and increase survival to the second or third decades of life. All these processes require accurate and reliable methods. 

Knowledge of the molecular basis of the disease in this family could help people at risk and improve genetic counseling. 
